# Raccoons Reveal Hidden Diversity in Trabecular Bone Development

**DOI:** 10.1093/iob/obae038

**Published:** 2024-10-21

**Authors:** T Reinecke, K D Angielczyk

**Affiliations:** The Committee on Evolutionary Biology, University of Chicago, Chicago, IL 60637, USA; Negaunee Integrative Research Center, Field Museum of Natural History, Chicago, IL 60605, USA

## Abstract

Trabecular bone, and its ability to rapidly modify its structure in response to strain exerted on skeletal elements, has garnered increased attention from researchers with the advancement of CT technology that allows for the analysis of its complex lattice-like framework. Much of this research has focused on adults of select taxa, but analysis into trabecular development across ontogeny remains limited. In this paper, we explore the shift in several trabecular characteristics in the articular head of the humerus and femur in *Procyon lotor* across the entirely of the species’ lifespan. Our results show that while body mass plays a role in determining trabecular structure, other elements such as bone growth, increased activity, and puberty result in trends not observed in the interspecific analysis of adults. Furthermore, differences in the trabeculae of the humerus and femur suggest combining distinct boney elements in meta-analysis may obfuscate the variety in the structures. Finally, rates at which fore and hindlimb trabeculae orient themselves early in life differ enough to warrant further exploration to identify the currently unknown causes for their variation.

## Introduction

The bony portion of the skeletal system that comprises the internal framework for most vertebrate species is comprised of two types of tissue: cortical and cancellous bone. The cortex forms the rigid and smooth dense outer layer of bone, whereas cancellous bone is internally arranged as a complex web of bony struts surrounded by bone marrow. These struts, commonly known as trabeculae, typically orient in the direction of primary strain to increase the overall strength of the bone with a diminished weight when compared to cortical structures (Wolff 1892; Koch 1917; Fyhrie and Carter 1986). The direction and magnitude of strain exerted on bone can vary widely throughout an individual's life, and trabeculae have evolved to rapidly adapt to these changes. As these struts are individually small and are surrounded by red marrow rich in osteoblasts and osteoclasts responsible for bone modification, trabeculae have been demonstrated to reorient and change shape in a period of weeks to months, a process that would take cortical bone up to 10 times as long (Parfitt 1988; Morgan et al. 2013). Due to its ability to rapidly adjust its structure, trabecular bone has become an important model for understanding the ways in which bones remodel in response to changes in loading strain.

Advancements in high-resolution computed tomography scans and computational analysis over the last few decades have allowed researchers to better describe and interpret complex trabecular structures, and numerous studies of cancellous bone in humans and our primate relatives have been undertaken. These studies have explored variation across trabecular characteristics in several load-bearing bones (e.g., vertebrae, femur, tibia) and the ways in which cancellous bone scales with body size (Rafferty 1998; MacLatchy & Müller 2002; Doube et al. 2010; Ryan and Walker 2010; Barak et al. 2017; Tsegai et al. 2018). Studies that have focused on the effects of ontogeny in primates have identified a trend toward increasing bone volume fraction and thickness as juveniles develop the locomotor mechanics they will utilize as adults (Saers et al. 2022A; Ryan et al. 2017; Tsegai et al. 2018). Humans demonstrate a sharp decrease in these values during their first year, likely caused by the delay in locomotor transitions compared to our closest relatives (Ryan and Krovitz 2006).

Although research on humans and primates has highlighted applications for biomedical work and models for understanding the evolution of bipedality (Ryan and Krovitz 2006; Ryan et al. 2017; Saers 2017; Tsegai et al. 2018; Saers et al. 2022A), there have been far fewer publications released focused on other mammalian clades. Primates are a less-than-optimal clade to use as a basis for extrapolating trends in other mammals as their adaptations toward specialized grasping arboreal behaviors have resulted in limb structures and hindlimb dominance that stand out as distinct to many other mammals (Larson et al. 2000; Young 2012). Humans are an even poorer model as our unique bipedal stance limits the use of our forelimbs in regular locomotory behavior. It should come as little surprise, then, that analysis of trabecular bone in humans and primates focuses primarily on the hindlimb and vertebral column (Nottestad et al. 1987; Rafferty 1998; Fajardo and Müller 2001; Morgan and Keaveny 2001; MacLatchy and Müller 2002; Ryan and Krovitz 2006; Fields et al. 2011; Wang et al. 2015). Additionally, advancements in medicine and quality of life have extended human lifespans far beyond the average range seen in pre-civilization individuals, resulting in trabecular changes in later stages of life that are frequently driven by diseases such as osteoporosis, rather than the standard effects induced solely by aging (McDonnel et al. 2007; Chen et al. 2013). Although studies of non-primate taxa have been conducted (Tanck et al. 2001; Amson et al. 2017; Mielke 2018; Smith and Angielczyk 2020; Zack et al. 2023), these analyses often sample small and/or autapomorphic clades or do not consider the potential effects of ontogenetic variation. Our understanding of trabecular development primarily stems from primate clades with a distinct brachial locomotor system and limb pair dominance compared to other mammals (Demes et al. 1994; Schmitt and Hanna 2004; Young 2012), but it is possible the trabeculae of other species differ in their development, especially given differences in which pair of limbs endures more strain during locomotion. Recent research has sought to investigate ontogenetic trabecular development in Japanese macaques as a model for a terrestrial quadrupedal ecomorphotype (Saers et al. 2022A), but more work will be necessary to analyze differences present in long limb elements, and to identify trabecular growth trends unique to primates. As trabecular research continues to expand in scope, identifying sources of potential trabecular variability will become integral in better understanding its diversity across a wide spectrum of taxa.

This study seeks to explore the development of trabecular structures in the articular heads of both the humerus and femur across the ontogeny of a less derived mammal species: *Procyon lotor* (raccoon). Raccoons were selected for two key reasons. First, they are one of the few species with a robust, non-destructive age-determination system that facilitates precision sampling of individuals across a full range of ages and ontogenetic stages (Grau et al. 1970). Second, as a prevalent mid-sized mammal present throughout much of North America, the volume of raccoon specimens housed within collections ensures that a large and diverse assemblage of individuals from a specific region can be sampled.

Based on observations in primates and *Sus domesticus*, we predict a significant difference in the trabecular characteristics between the humerus and femur of raccoons over the course of ontogeny (Tanck et al. 2001; Tsegai et al. 2018; Saers et al. 2022B). We also predict a significant difference in the trabecular architecture of the femur and humerus of adult raccoons due to several behaviors that exert higher strain on the hindlimbs. Adult raccoons often engage in vertical climbing that utilizes the hindlimbs as both a bracer and propeller, which, in turn, places a greater strain on the femur (Hanna et al. 2017). Raccoons also regularly utilize a bipedal posture to both forage for and carry food, often to a river where it can be washed before consumption (MacClintok and Thomas 1981; McClearn 1992). Juvenile raccoons younger than 12 weeks of age who have yet to leave the den and practice these more strenuous hindlimb-dominated behaviors will likely feature a diminished difference in fore and hindlimb trabeculae as all four limbs are utilized more consistently. Additionally, a lack of specific high-strain locomotor behaviors will likely result in more variable trabecular primary orientations in these pre-pubescent raccoons. Finally, following trends seen in other taxa, we predict trabecular bone volume fraction and anisotropy to increase with age as individuals grow and develop (Tanck et al. 2001; Saers et al. 2022A).

## Methods

Fifty-five wild specimens of raccoon (*P. lotor)* native to the Great Lakes region were sampled. The skeletal remains of all specimens were housed at the Field Museum of Natural History and the Illinois State Museum. Only wild specimens were selected as limited mobility, behavioral variance, and other factors have been demonstrated to have a measurable effect on trabecular structures of captive specimens (Chirchir et al. 2022; Zack et al. 2022). Individuals were aged based on the presence or absence of several skull sutures following Junge and Hoffmeister (1980), an approach that has been demonstrated to have a high level of accuracy for both sexes compared to other non-destructive methods (Grau et al. 1970; Fiero and Verts 1986; Supplemental Table S1). Each specimen was assigned to one of the five age groups: 2 months (*n* = 13), 4–12 months (*n* = 11), 14–24 months (*n* = 10), 26–38 months (*n* = 7), and 46 + months (*n* = 14). The gaps between age groups are a part of Junge and Hoffmeister's (1980) approach and are retained here. These age groups separate several stages in raccoon ontogeny, with juveniles beginning to walk around their nests by 2 months, and individuals reaching sexual maturity around 12–13 months of age (MacClintock and Thomas 1981). The exact timing of the loss of the cartilaginous growth plates and the fusion of the epiphyses, signaling the end of elongation of the limb bones, varies among individuals, but typically occurs between the first and second years of life (Johnson III 1969). Age groups past 2 years were segregated to determine the effect increasing body mass and age-related wear and tear may have on trabecular structures.

The right humerus and femur of each specimen were selected for analysis. Any bones that featured damage to the proximal articular head or morphological features affected by disease were excluded. If a specimen lacked a viable element from the right side, the corresponding left element was mirrored to replace it. This mirroring was only necessary in a small number of cases: four of the humeri and seven of the femora.

Specimens were scanned using a GE Phoenix V | tome | x μCT (micro-computed tomography) scanner with a 240 kV micro-focus X-ray tube at the University of Chicago's PaleoCT facility (RRID:SCR_024763). Each scan was conducted to capture the entire morphology of upper limb elements, with multi-scans conducted for larger specimens. Scan resolution ranged from 24.0375 to 46.937 µm, with smaller specimens requiring a higher resolution to preserve all material (Supplemental Table S1). Within each age group, resolutions varied by no more than 10 µm. Scans were reconstructed in GE Phoenix datos | x, and image stacks were aligned and cropped in VGStudioMAX 3.3.

**Fig. 1:**
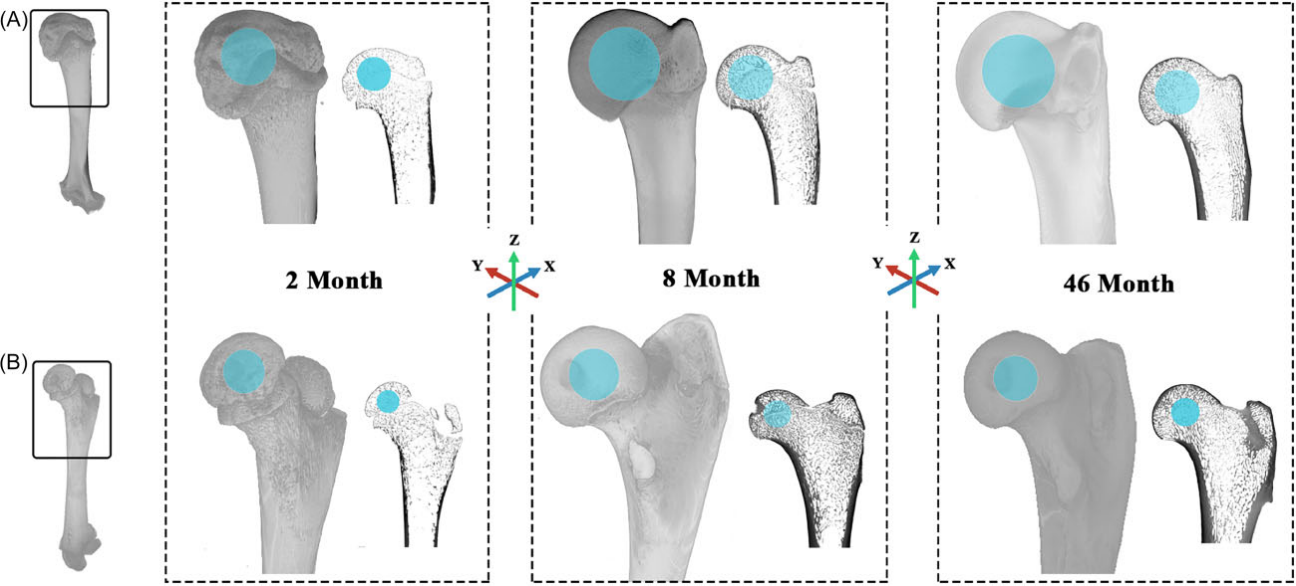
Standardized orientations for the Humerus (A) and Femur (B) at various ages. The spheres highlighted within each bone represent the sampled ROI. Cross-sectional views along the X–Z plane are also provided.

Scans were modified in the image processing program Dragonfly 2022.2 (Dragonfly 2022) so that each bone was uniformly oriented. First, the midpoint of the distal and proximal metaphyses was aligned along the *Z*-axis. Second, the lateralmost points where the proximal articular head contacted the anatomical neck were aligned along the *Y*-axis such that the articular surface pointed in the −X direction, and the tubercles point in the +X direction (Fig. 1). This orientation does not reflect how an animal would hold their proximal limb elements in life, but rather reflects a uniform orientation for comparison between individuals that can be replicated for other species as well. Previous studies have largely ignored the need for a standard orientation of the entire element due to the exclusion of trabecular orientation measurements and have instead used landmarks of a specific element (Barak et al. 2011) or the midsection of anatomical planes relative to the articular head (Ryan and Ketcham 2005) to determine region of interest (ROI). Those that have included specific orientations for long-limb elements have used similar methods to orient the bones vertically but rely on taxon-specific features for its rotation (Amson et al. 2017; Mielke et al. 2018). Our approach in this study has been designed to facilitate comparative analysis of disparate limb elements. Additionally, because these methods utilize the overall shape of the bone, rather than relying on taxonomic-specific landmarks, this methodology can be used for comparisons between taxa in an interspecific analysis.

Once bones were oriented, the articular head was isolated within a box whose sides contacted the innermost edges of the cortical bone. A spherical ROI was expanded out from the center of this box to include the maximum number of trabeculae while excluding any cortical bone (Supplementary Fig. S1 for a visual representation of the orienting and ROI selection process). This ROI was segmented using an Otsu sorting algorithm (Otsu 1979), which has been shown to usually preserve small trabecular structures and remove free-floating particles while avoiding the overestimation of bone volume fraction (Smith and Angielczyk 2020).

The volume of the segmented trabeculae was compared in Dragonfly to the volume of the spherical ROI to determine bone volume fraction (BV/TV). Two binarized TIFF-stacks, one of the trabeculae and one of the non-trabecular spaces, were imported into ImageJ for further analysis (Schneider et al. 2012). Using the plugin BoneJ (Doube et al. 2010), each TIFF-stack was purified (i.e., small floater particles are removed) before being processed to determine anisotropy (DA), trabecular thickness (TbTh), and trabecular spacing (TbSp). These stacks were then imported into Quant3D (Hoebeke and Trubuil 1999) where trabecular number (TbN) was measured, and the primary trabecular eigenvectors were identified; the latter were then converted into azimuth and plunge using code developed by Amson et al. (2017). The mean intercept length tensor was selected to determine the fabric tensor as it has been demonstrated to more accurately predict the mechanical properties of trabecular bone compared to other methods tensors (Zysset 2003; Cowin and Doty 2007; Moreno et al. 2014).

Log-transformed body mass estimates for each specimen were calculated using the circumferences of the humerus and femur and the scaling equation of Campione and Evans (2012). As various trabecular structures have been demonstrated to scale with body mass in several mammalian species (Barak et al. 2013; Kim et al. 2017; Mazzetti et al. 2017; Saers 2017; Zack et al. 2023; Smith et al. 2024), several regression plots were constructed to assess the relationship between trabecular characteristics and body size. K-fold cross validations were utilized to determine which degree featured the lowest mean square error for each of the polynomial regressions. The predicted linear regression slopes were also estimated for each characteristic and varied depending on their units. BV/TV and anisotropy are unitless ratios with an isometric slope of 0. TbN is measured as individual struts per mm and has an expected isometric slope of −1/3. TbTh and TbSp are both linear measurements and have a predicted isometric slope of 0.33 as these features scale at 1/3 relative to volume (Mielke et al. 2018; Plasse et al. 2019; Smith et al. 2023).

Pearson's correlation coefficients were calculated between every univariate trabecular characteristic in both the humerus and femur using the pcor function of the ppcor package in R (Kim 2015). The correlation coefficients were used to identify the presence and strength of any potential linear correlation between features. The variability of the trabecular bone metrics was compared using the coefficient of variation. This statistic expresses standard deviation as a proportion of the mean, making it appropriate for comparing metrics with different absolute magnitudes. The coefficient of variation was calculated using the CV function as a part of the raster package in R (Hijmans 2023).

A principal component analysis (PCA) was performed on the estimated body mass, TbN, spacing, thickness, density, anisotropy, and trabecular orientation for the femur and humerus of each specimen. A PCA plot was calculated with the raw values of all trabecular characteristics save for azimuth, whose values were transformed using the formula $| {| {x - 180} | - 90} |$. This transformation was used to more closely pair nearby angle measurements with differing angle values (e.g., 359° and 1°) and to quantify trabecular azimuth as pointing somewhere along the spectrum between the anterior/posterior and medial/lateral planes. Each PCA analysis was performed using the prcomp function as part of the stats package in R (R Core Team 2023).

Because several trabecular characteristics featured a non-normal distribution that could not be transformed, nonparametric Kruskal–Wallis (Kruskal and Wallis 1952) and Dunn tests (Dunn 1964) were utilized in R using the Kruskal test (R Core Team 2023) and Dunntest (Dinno and Dinno 2017) packages respectively. The aims of these tests were to determine if the means for each trabecular characteristic were significantly different over the course of the species’ ontogeny. If the results of Kruskal–Wallis test of a characteristic was significant at *α* = 0.05 significance level, a Dunn test was run to determine which age group means were significantly different.

## Results

**Fig. 2:**
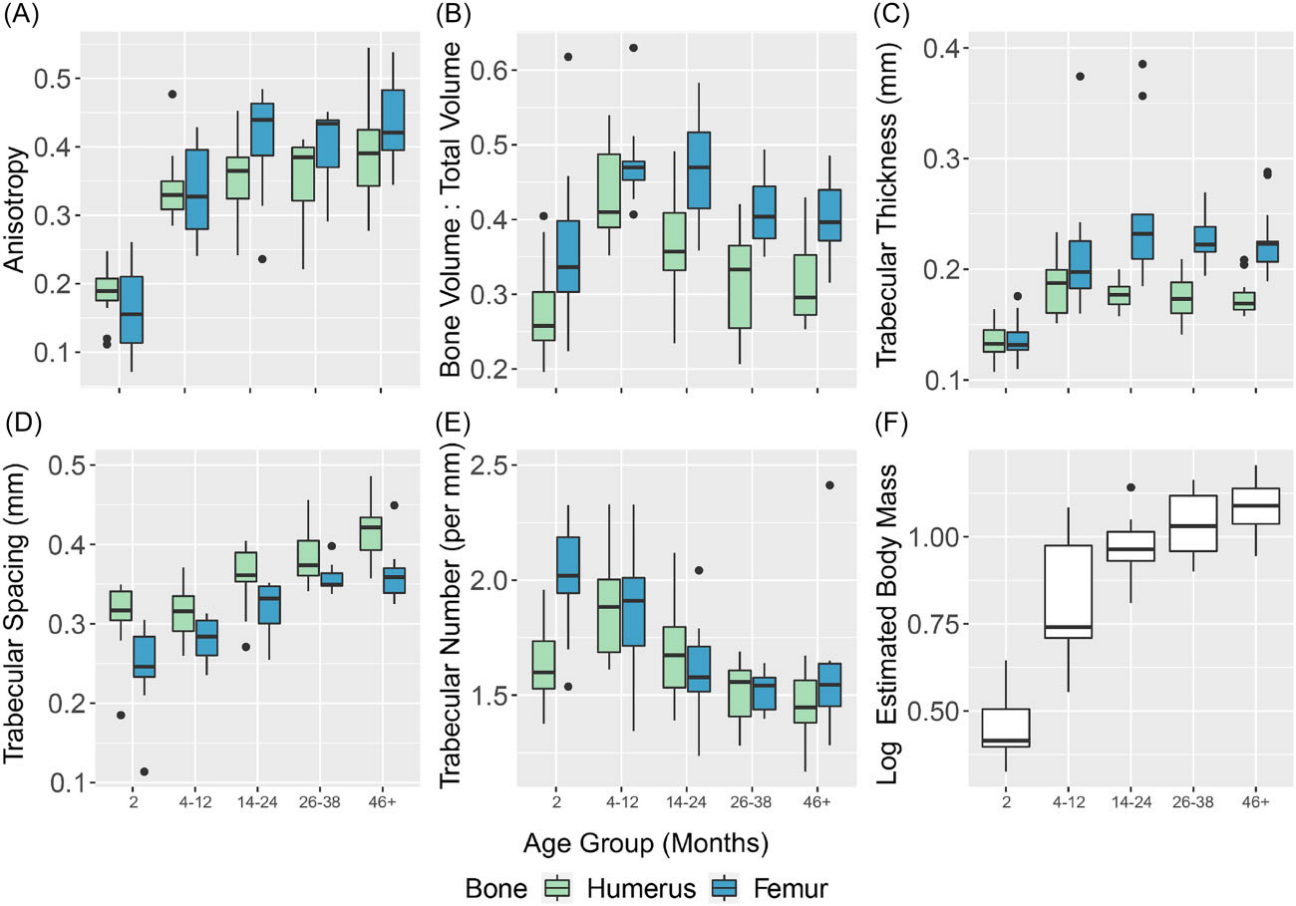
Violin plots for each trabecular characteristic in the femur and humerus (A–E) and estimated log-body mass (F).

**Fig. 3:**
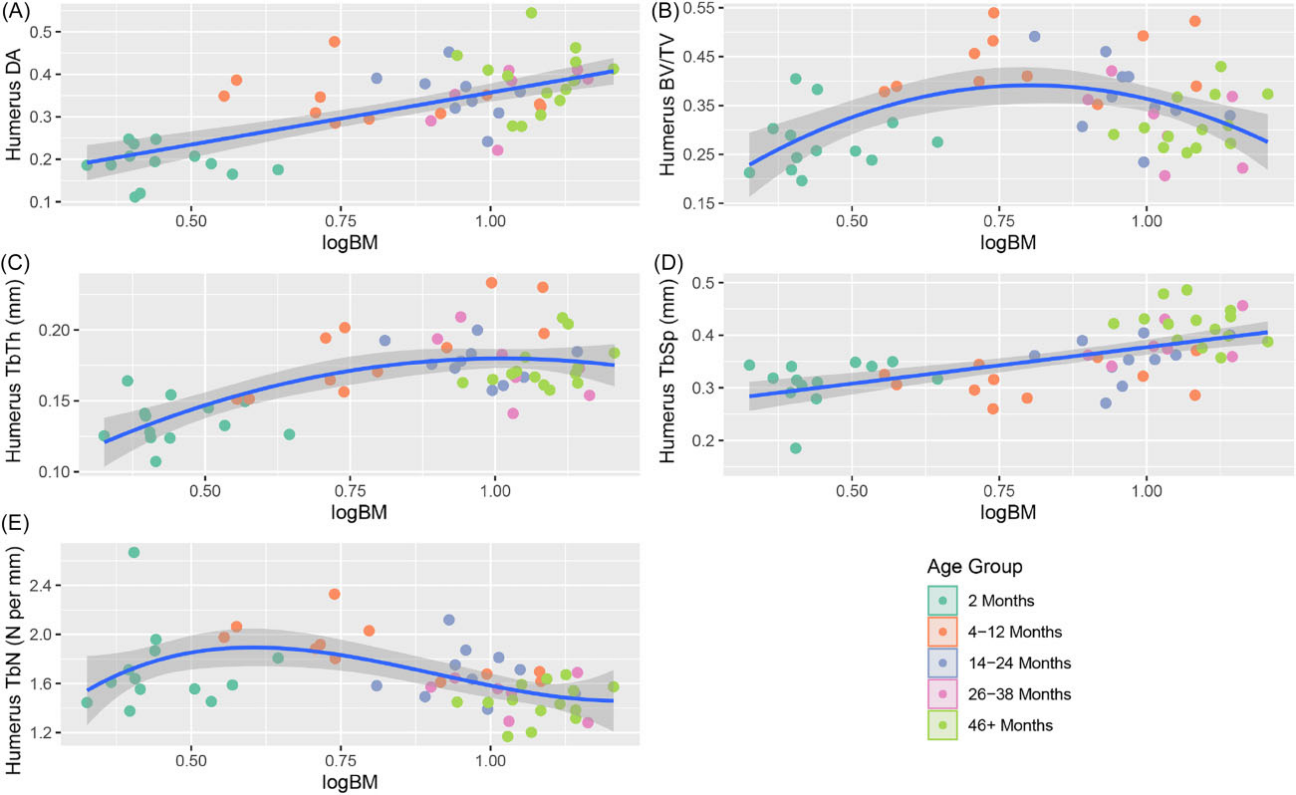
Regression plots of the trabecular characteristics of the humerus v log corrected body mass estimates. The characteristics in order are DA (A), BV/TV (B), TbTh (C), TbSp (D), and TbN (E).

**Fig. 4:**
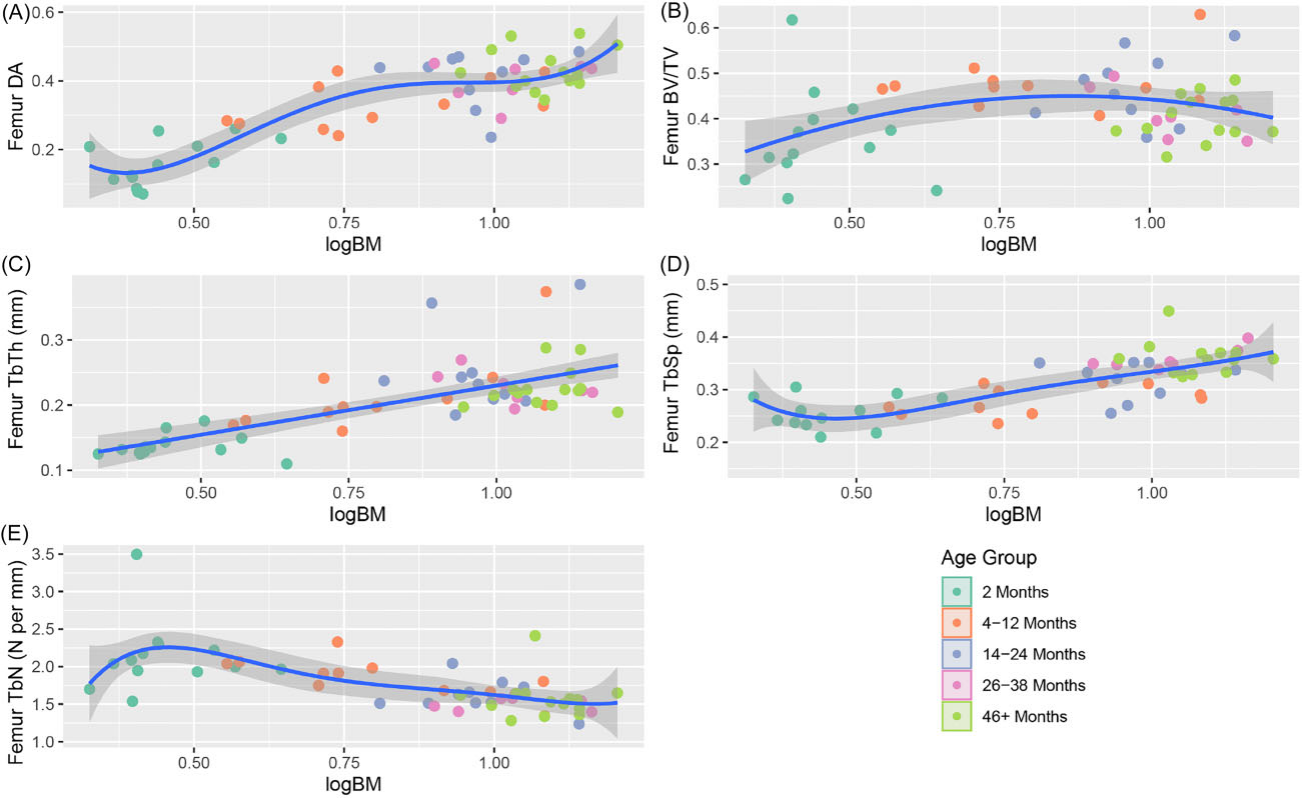
Regression plots of the trabecular characteristics of the femur v log corrected body mass estimates. The characteristics in order are DA (A), BV/TV (B), TbTh (C), TbSp (D), and TN (E).

When the raw trabecular characteristics are divided into age bins, we observe a consistent pattern in which all metrics for 2-month raccoons except femoral TbN are statistically lower than for older age groups (Table 1; Fig. 2). Both bones demonstrate a peak in BV/TV values within the 4–12 month groups, before gradually decreasing for all subsequent age groups. In the case of the 46+ group, femoral BV/TV values are nearly comparable to those of the 2-month group. Trabecular number is also unique in that it displays the highest disparity between the humerus and femur in 2-month individuals, with the values becoming more comparable between the fore- and hindlimbs by 8 months. From 4 months onward, trabecular values either approximate a plateau or form a gradual logarithmic curve (Figs. 3 and 4). The coefficients of variation of the age groups indicate that for both the humerus and femur, 2-month raccoons present the highest spread for anisotropy, BV/TV, and TbN (Table 2).

**Table 1:** Raw trabecular values for each age group.

		Humerus					Femur				
Age group	Number of individuals	Anisotropy	BV/TV	TbTh (cm)	TbSp (cm)	TbN	Anisotropy	BV/TV	TbTh (cm)	TbSp (cm)	TbN
2 months	13	0.19	0.276	0.0135	0.0311	1.71	0.16	0.358	0.0137	0.0245	2.13
4–12 months	11	0.342	0.437	0.0185	0.0315	1.87	0.333	0.477	0.0214	0.028	1.86
14–24 months	10	0.355	0.369	0.0177	0.0354	1.69	0.411	0.468	0.0252	0.0319	1.62
26–38 months	7	0.351	0.314	0.0174	0.0386	1.51	0.399	0.412	0.0228	0.0358	1.52
46 + months	14	0.386	0.311	0.0174	0.0419	1.45	0.434	0.404	0.0226	0.0361	1.57

**Table 2:** Coefficents of variation for each age group.

		Humerus					Femur				
Age group	Number of individuals	Anisotropy	BV/TV	TbTh (cm)	TbSp (cm)	TbN	Anisotropy	BV/TV	TbTh (cm)	TbSp (cm)	TbN
2 months	13	22.1	22.7	11.3	14.1	19.5	42.1	29.1	12.9	20	21.8
4–12 months	11	15.6	14.6	15.8	10.8	11.8	20.7	12.2	27.6	9.61	13.9
14–24 months	10	16	20.5	7.59	11.8	12.6	19.5	16.4	26.1	10.9	13.2
26–38 months	7	20.2	25.2	13.3	10.8	10.7	14.6	13.1	10.6	5.77	6.14
46+ months	14	19.2	17.1	8.9	8.7	10.4	14	12.5	13.1	8.6	17.1

**Fig. 5:**
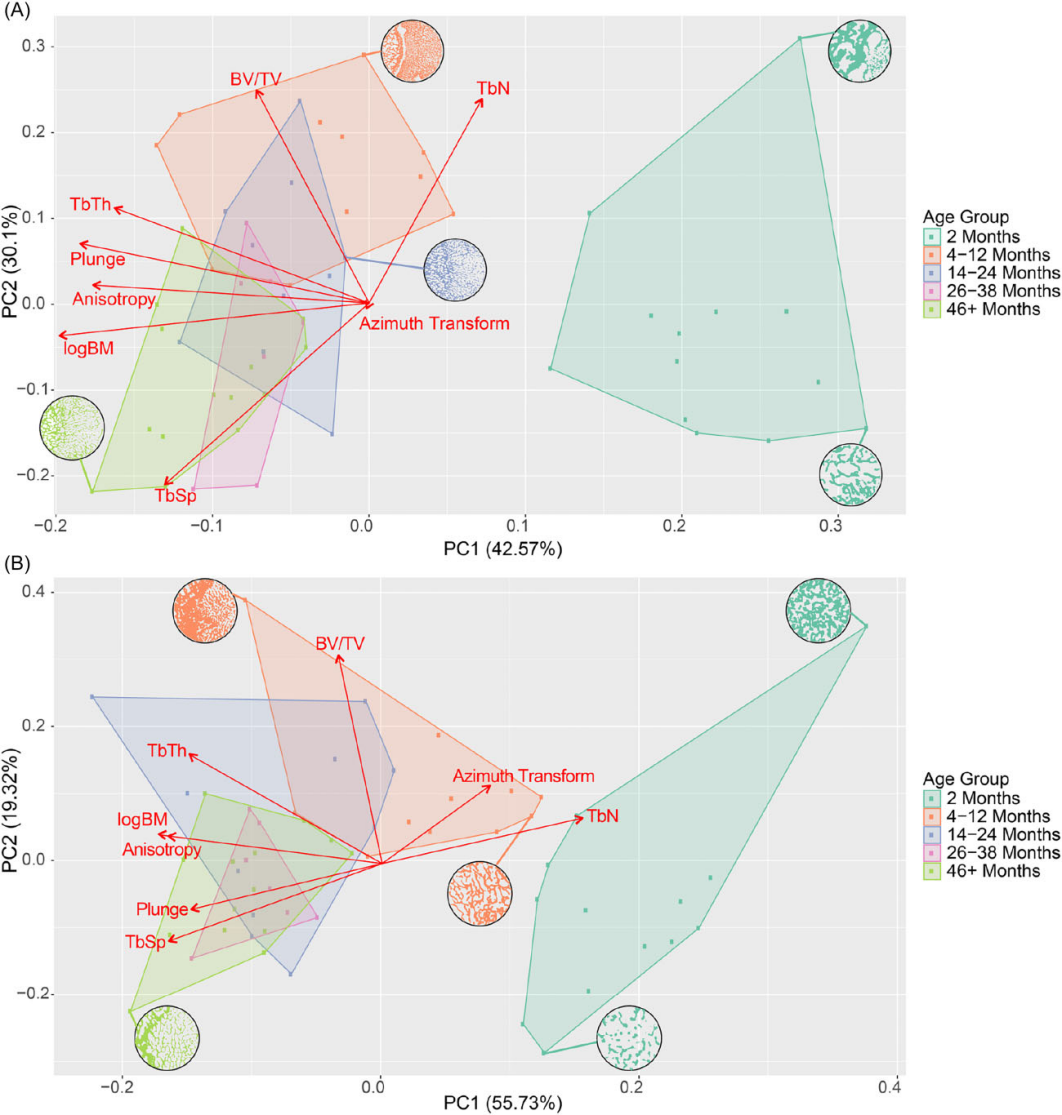
PCA and biplots plots of all trabecular characteristics and estimated logBM for both the humerus (A) and femur (B). PCA plots include a cross sectional view of the trabeculae of certain individuals taken from the center of the ROI.

Principal component analyses for both the humerus and femur feature a clear separation between the 2-month-old specimens and the other age groups, which cluster toward the left of the PCA plots (Fig. 5). For both limb elements, logBM, DA, and plunge receive strong loadings on the first PC, with all values decreasing as one move towards higher PC scores. Principal component two features more disparity in loadings between the humerus and femur, with only BV/TV featuring a strong loading in the positive direction in both cases. For the humerus, TbN, and TbSp have strong loadings in the positive and negative directions, respectively, whereas the femur only exhibits a mild loading for the transformed azimuth on PC 2.

Kruskal–Wallis tests on each trabecular characteristic determined that there was a statistically significant difference for the means of all femoral and humeral metrics with respect to age (Table 3). The Dunn tests (Dunn 1964) demonstrated that among all age groups, the 2-month age group most consistently demonstrated significant differences in trabecular metric means when compared to the older age groups; comparisons between the older age groups more frequently returned non-significant differences (Supplementary Table S2).

**Table 3:** Kruskal-Wallis p-values.

	Trabecular characteristic	*P*-value
Humerus	DA	4.22E-06
	BV/TV	0.0001006
	TbTh	3.11E-05
	TbSp	5.88E-07
	TbN	0.0002091
	Azimuth transform	0.7365
	Plunge	1.44E-06
Femur	DA	6.83E-07
	BV/TV	0.001033
	TbTh	2.21E-06
	TbSp	4.66E-08
	TbN	3.87E-05
	Azimuth transform	0.1063
	Plunge	9.15E-05
	logBM	6.74E-08

Although analysis of Pearson's correlation coefficient found nearly all trabecular characteristics to be significantly correlated with one another, only TbTh and BV/TV featured a “very strong” correlation in both the humerus and femur (absolute *r* value between 0.8 and 1; Evans 1996; Table 4). The next highest *r* value pair in both bones was DA with TbSp, with each bone featuring an *r* value above 0.7. Regression analysis indicated a significant relationship between all trabecular characteristics and body mass. Both the humeral (Fig. 3) and femoral (Fig. 4) trabeculae show similar trends as body mass increases, with DA, TbTh, and TbSp increasing with body mass, and BV/TV and TbN represented by concave parabolic arcs. For the humerus, the rate of increase in TbTh during the first year of life is only half that of TbSp before leveling out to a relative plateau at the end of puberty. This trend is not present in the femur where TbTh and TbSp growth rates remain relatively similar throughout all age groups. Despite this difference, both bones see a peak of BV/TV around 8–12 months, with the subsequent decrease at later ages likely stemming from a steady decline in TbN from the age of 4 months onward.

**Table 4:**
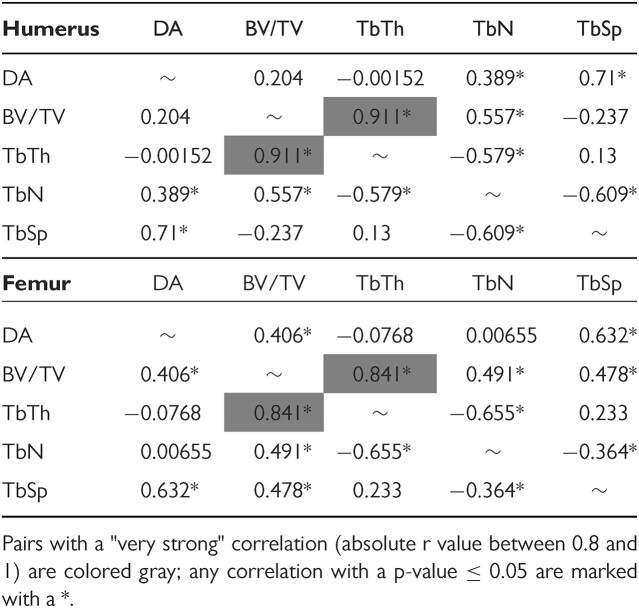
Pearon's correlation coefficents between trabecular characteristics.

**Fig. 6:**
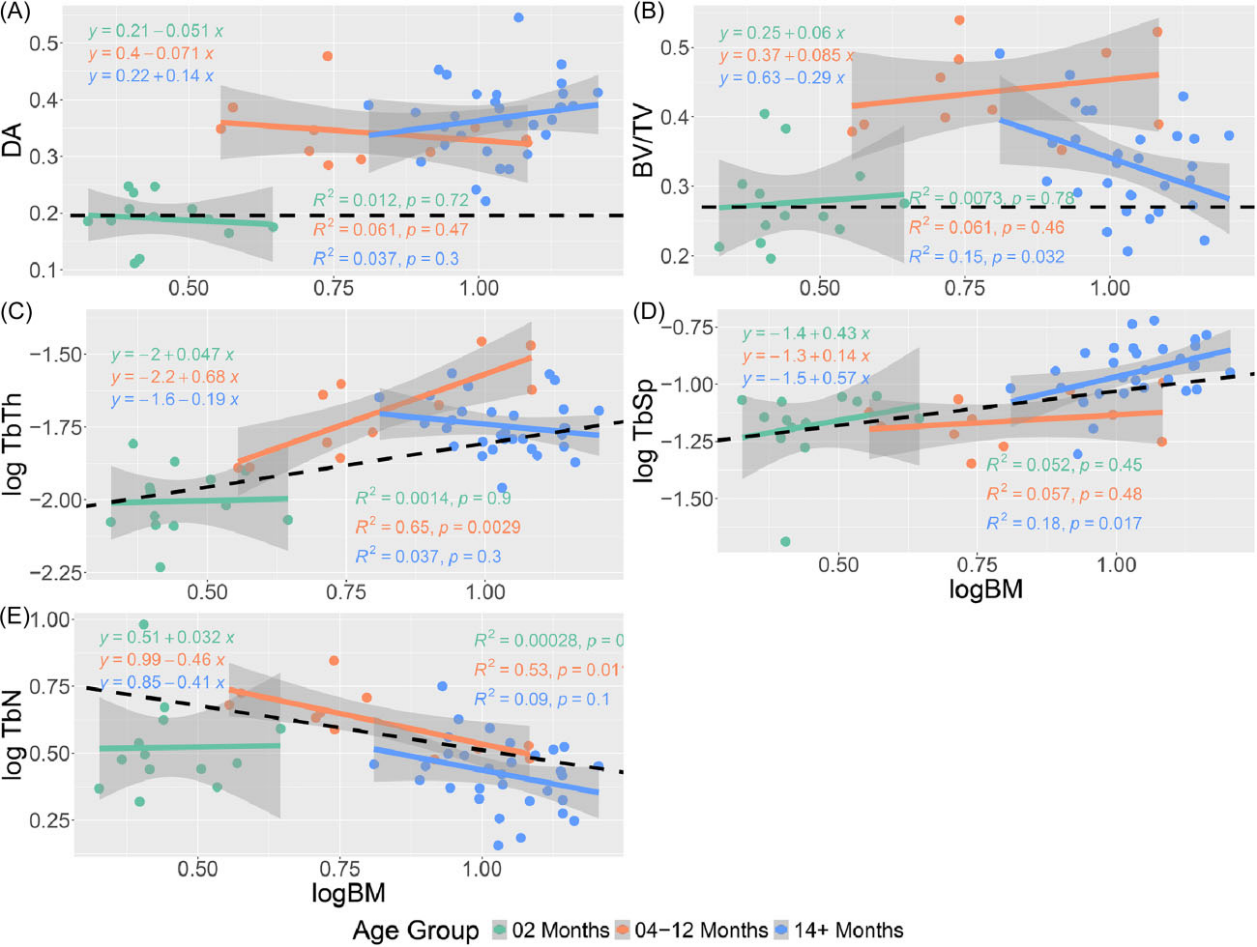
Regression plots for each trabecular characteristic of the humerus with regression slopes calculated for three age groups: 2 months, 4–12 months, and 14+ months. Groups were selected to identify trends in trabecular development before, during, and after puberty. The black dotted line represented the estimated regression slope with respect to isometry. The characteristics in order are DA (A), BV/TV (B), TbTh (C), TbSp (D), and TbN(E).

**Fig. 7:**
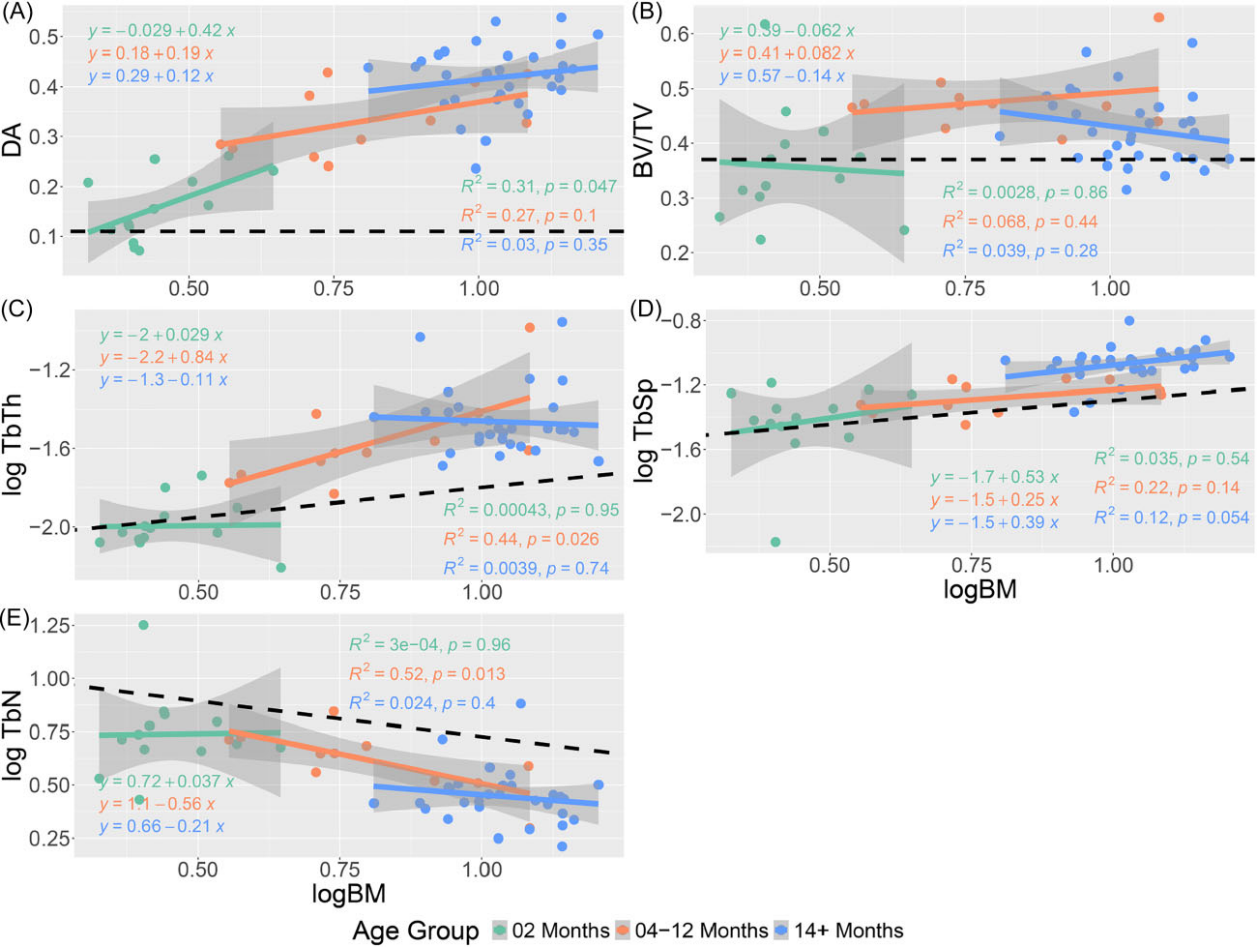
Regression plots for each trabecular characteristic of the femur with regression slopes calculated for three age groups: 2 months, 4–12 months, and 14+ months. Groups were selected to identify trends in trabecular development before, during, and after puberty. The black dotted line represented the estimated regression slope with respect to isometry. The characteristics in order are DA (A), BV/TV (B), TbTh (C), TbSp (D), and TbN(E).

Separating the raccoons into three distinct age bins (Figs. 6 and 7) highlights that a consistent scaling of each trabecular characteristic with body mass is not preserved in individual age groups. Of the 3 age groups, individuals 14 months and older show the most consistently allometric trends, and often with negative gradients as opposed to the positive slopes seen in younger age bins.

**Fig. 8:**
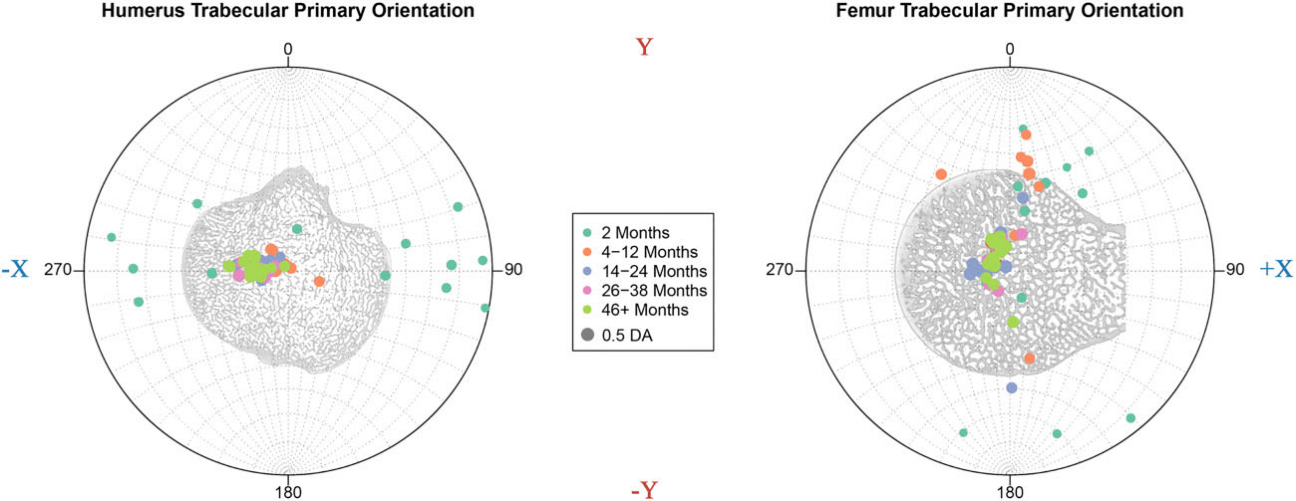
Primary trabecular orientations of the trabeculae in the humerus and femur for each *Procyon lotor* individual. Points are color coded with regards to age groups, and the size of each point reflects anisotropy, with larger points representing higher anisotropic values (DA). Cross sectional views of both the humeral and femoral articular heads are included to illustrate the orientations of each bone. The X and Y axes are included in the margins to match those presented in Fig. 1. The orientations of each bone do not reflect how the bone is held in life, but rather reflect a uniform orientation that makes direct comparison between the two more pronounced.

When representing the primary trabecular orientation via stereomorphic projection (Fig. 8), 2-month individuals again display values that notably deviate from the other age groups. Specifically, the orientations for 2-month raccoons trend toward very horizontal directions, with trabeculae in the humerus and femur aligned with the anterior/posterior axis for each bone. Although the trabeculae rapidly converge toward a nearly vertical orientation in the center of the stereomorphic projection as the raccoons age, there seems to be a lag in the femur, with raccoons in the 4–12 month age range still featuring horizontal trabecular orientations, albeit more vertical than younger individuals.

## Discussion

### Trabecular characteristics across ontogeny

In this analysis, we quantified trabecular bone architecture in the proximal articular head of the humerus and femur across an ontogenetic series of raccoons ranging in age from 2 months to over 46 months. One of the most striking aspects of the results presented here is the consistent difference in most aspects of trabecular bone structure between the 2-month-old raccoons and the older individuals in the dataset. At 2 months of age large portions of the long limb bones are still undergoing endochondral ossification, a process that forms and arranges chondrocytes before activating regional cell death to prepare nearby cartilage to calcify into bony structures (Mackie et al. 2008). By 8 months, the ossification process has replaced most of the hyaline cartilage with bone, resulting in only a thin layer of cartilage surrounding the articular head and an internal epiphyseal growth plate that will fade between the first and second year (Johnson III 1969; Fig. 1). This cartilage, while still operating as a shock absorber and distributor for strain strong enough to influence trabecular development (McKinley and Bay 2001), does not deflect the same magnitude of strain on the bony articular head itself as the original cartilaginous growth plate. Although the effect of ontogenetic cartilaginous development on trabecular structures has not been formally tested, it is logical to hypothesize the diminished volume of soft tissue buffer drives the trabeculae towards a larger, denser, and more anisotropic arrangement in response to higher locomotor stresses.

It is also important to note the presence of an epiphyseal growth plate in younger raccoons that is formed during endochondral ossification and is gradually remodeled as the cartilage is replaced by bone (Grau et al. 1970; Wheeler 1975; Reno et al. 2007). In raccoons, these plates are present within the articular head of both the humerus and femur, and our ROIs include these structures. Given that the plates act as bracing points for trabecular attachment, we might expect trabeculae for younger individuals to display higher DA and thickness values than adults who have lost this plate. However, our results suggest that these characteristics are significantly lower in younger individuals (Fig. 2), with trabecular becoming thicker and more uniformly oriented as the plate begins to be remodeled. Therefore, we posit that the plate's impact on the trabeculae of the articular head is minimal and does not impact the results of this paper. The specific impact of the epiphyseal plates on trabecular growth is important to quantify, and we are hopeful that future research examining ontogenetic development in other mammal clades will delve more deeply into this topic.

Although 2-month-old raccoon cubs have developed enough muscularity to allow small excursions around their den, it has only been 1 week or 2 since these exploratory outings began (MacClintock & Thomans 1981). Trabeculae have been demonstrated to undergo rapid remodeling in response to stress (Barak et al. 2013), but a period of only 1–2weeks is not long enough to have facilitated the shift in trabeculae to match the structures present in older individuals, consistent with observations over ontogeny in other species (Tanck et al. 2001). More than just time, the sporadic low-stress behaviors of which these juveniles are capable at such a young age would have a weaker effect on trabecular development when compared to behaviors with high magnitudes of loading in which adults engage, such as climbing, carrying food, and rearing up on hind legs (McClearn 1992; Ruff et al. 2006).

After a several more weeks of increasingly long trips from the den, raccoon pups are fully weaned and left to explore the world on their own. It is possible that their increased reliance on high-stress foraging behavior may lead to a rapid development of trabecular architecture more in line with structures present in older adults. Additionally, the loss of substantial hyaline cartilage may ensure that the direction and intensity of loading strain on the bone itself is more in line with that seen in sexually mature adults.

When looking broadly at trabecular trends across the entire range of ontogeny (Fig. 2), nearly all follow a similar trajectory to body mass: A rapid increase from the 2-month age group to the 4–12-month bin before gradually settling along a horizontal trend. Among this clustering of trabecular characteristics, only DA features values that consistently increase with age for both the humerus and femur. Trabecular spacing is the sole characteristic to appear to steadily increase at a relatively consistent rate for both the humerus and femur over the course of a raccoon's life. The reason for this linear increase, especially in relation to other characteristics, is unclear, but this steady trend of increasing porosity within the bone appears to drive struts to reach a maximum thickness before decreasing the number of struts found within a sampled volume. This decrease in strut number, paired with increased strain exerted on the long-limb elements and a decline in bone's ultimate stress with age (Keaveny et al. 2001), appears to drive struts towards a more uniform directionality. However, despite the linear increase of TbSp, the asymptotic DA graph suggests that there is a maximum uniformity in the direction of trabecular struts that still allows for enough disparity to support the range of loading strains exerted on the long-limb elements. Future research is needed to assess if a DA asymptote of 0.5 is present in other species and niches, or if this maximum varies across a wide spectrum of taxa and ecomorphotypes.

The development of BV/TV and DA across ontogeny in raccoons compared with previously tested species shows a progression more in line with *Sus* than primates. BV/TV in both quadrupedal species peaks during puberty before gradually decreasing through adulthood, whereas primates display a sharp decline in BV/TV during their pre-locomotor ages before steadily increasing toward a plateau in adulthood. Trabecular anisotropy in *Procyon* and *Sus* also increases rapidly during puberty and tapers off into adulthood, whereas chimpanzee trabeculae displays a concave pattern across ontogeny that reaches a low around 6 years before returning to comparable levels as newborns (Tanck et al. 2001; Tsegai et al. 2018; Saers et al. 2022B). This disparity in trabecular growth can be attributed to a variety of potential factors. These include differences in posture between quadrupedal and brachial species, variations in maturation rates, and phylogenetic position. Further analysis will need to independently explore each of these factors to identify, which have the most notable impact.

### Correlations between trabecular characteristics

A previous meta-analysis of correlations between trabecular characteristics identified relationships between TbTh and BV/TV, and TbN and BV/TV, with both featuring positive regression slopes in humans, mice, and rats (Barak et al. 2013). Other research by Ryan and Shaw (2012) further highlighted the interrelated nature of many trabecular characteristics and suggests that these features should be analyzed as a suite of traits that directly influence one another. Our analysis of the humerus and femur identified a strong correlation between TbTh and BV/TV in each bone independently, but only a moderate correlation between TbN and BV/TV. Much of this disparity may be attributed to changes that occur over ontogeny, with both TbTh and BV/TV reaching a maximum within the 4–12 month age range, whereas TbN steadily decreases with age.

The positive linear regression slope between TbTh and BV/TV within the humerus was congruent with values found in rats and mice, and higher than the values seen in humans (Barak et al. 2013). However, the same analysis conducted on femoral trabeculae resulted in a slope less than half that seen in the humerus, and significantly lower than the aggregate slopes of rats, mice, or humans. Given the wide disparity in correlations between TbTh and BV/TV within different bones, future research should be conducted to fully assess the diversity in trabecular architecture within commonly sampled bones (vertebrae, femur, humerus, tibia, and ulna) and regions of said bones (vertebral body, articular head, midshaft, and condyles). Additionally, meta-analyses of trabecular properties should prioritize isolating different bones from one another because signals may not be consistent across elements.

### Trabecular characteristics and body mass

Numerous studies have shown a correlation between trabecular architecture and body mass, especially for trabecular thickness and number (Doube et al. 2011; Barak et al. 2013; Kim et al. 2017; Shin et al. 2019). Our findings corroborate this relationship, with regressions indicating that every trabecular characteristic in both the humerus and femur is impacted by overall body mass (Mielke et al. 2018; Plasse et al. 2019; Smith et al. 2023). However, previous studies have indicated linear trends between trabecular structure and body mass, with the majority of these investigations focusing exclusively on adult individuals. The few that have sampled other age groups have focused exclusively on Hominini and have relied on small sample sizes due to a difficulty in acquiring juvenile specimens (Ryan and Krovitz 2006; Tsegai et al. 2018).

A few notable characteristics display a positive trend with regards to body mass in our dataset: DA and TbSp in the humerus, and DA, TbTh, and TbSp in the femur. In contrast, the remaining characteristics see a peak at a relatively young age, typically 4–12 months, before gradually decreasing for the remaining older individuals. The parabolic nature of these regression slopes can be explained by the interplay of other trabecular characteristics. BV/TV in both the humerus and femur sees a rapid increase in tandem with trabecular struts increasing in thickness through the first year of life. Starting at a logBM value of 0.75 (∼5.62 kg), a mass primarily presented by individuals within the 4–12 month age group, the change in strut thickness plateaus, whereas space between struts continues to increase and TbN steadily declines. Eventually, this widening of space between struts results in an overall BV/TV value by 3–4 years of age that is comparable to that seen in 2-month-old individuals.

Despite the allometric trends in trabecular characteristics across ontogeny, individual age bins are primarily in line with estimated isometric growth rates (Figs. 6 and 7). In both the humerus and femur, individuals in the 4–12-month age group who are old enough to have left the den retain a higher number of trabecular struts than predicted. Given that raccoons are generalist species who engage in a wide range of behaviors, the preservation of a higher number of struts may serve to act as a buffer against variable strains. Comparisons with other analyses places the slope for racoon TbN with respect to body mass as lower than other species (ranging from −0.21 to −0.56 compared to −0.146, respectively; Barak et al. 2013), although the interspecific slope was measured using adults only, rather multiple age groups than within a single species. Further analysis of the ontogenetic development of other species is needed to determine if there are measurable differences in rate of strut loss between species with respect to body mass when compared to intraspecific analyses.

Beyond TbN, the humeri of 4+- month old raccoons also display a significantly different rate of change in TbTh with isometry. Unlike TbN, which retained a negative allometric rate of change during and after puberty, the thickness of humeral struts appears to reach a peak around 1 logBM and 12 months of age, before decreasing throughout the remainder of an animal's life. This trend is once again at odds with interspecific trends, which show a steady positive correlation with body mass (Barak et al. 2013). Our results suggest that raccoons undergo a process of significant trabecular thickening through puberty. Once individuals have reached sexual maturity and the need for rapid bodily growth is diminished, the body begins to gradually reduce the thickness of struts to reduce density and maximize strut efficiency.

It is once again important to note that in comparing the slope of these raccoon age bins to interspecific trends presented by Barak et al. (2013), the latter values are based on a meta-analysis encompasses a variety of skeletal elements. The present scope of trabecular research leaves humans as the primary point of comparison for ontogenetic trends in trabecular architecture, but *Homo sapiens* have their own caveats that complicate comparisons with other species. Beyond the obvious differences in locomotion, lifestyle, and size, significant changes in trabecular architecture in older humans seem to be primarily driven by bone-related illnesses such as osteoporosis that affect reabsorption rates and trabecular production to a greater extent than diminished loading strain (Ryan and Krovitz 2006; McDonnel et al. 2007). The wild raccoon specimens used in this study showed no evidence of any bone pathologies and were likely unable to survive long enough in their natural habitat to develop these illnesses. As such, the gradual shifts in trabecular structures across ontogeny cannot be explained by the same causes as in humans. Additionally, ontogenetic studies of humans- focused primarily on the very young (Ryan and Krovitz 2006) or elderly (McDonnel et al. 2007), with limited research conducted on the pubescent age range where the most significant changes appear to occur in raccoons. Finally, there has been no real analysis of the development of the trabeculae of human humeri, which makes any direct comparisons to raccoon humeri problematic due to the noticeable differences in the trabecular characteristics of the fore and hind limb, to say nothing of how much more disparate these values likely would be in a bipedal species like *Homo sapiens.*

### Humeral and femoral disparity

Though both the fore and hindlimb elements show similar trends in the development of trabecular features across ontogeny, the frequent disparity in absolute values of the metrics between the two bones suggests a difference in loading strain. Most non-primate mammal clades are defined by a “forelimb driven” quadrupedal gait, in which the forelimbs experience a stronger substrate reaction force (Demes et al. 1994; Schmitt and Hanna 2004; Young 2012). This loading pattern would lead to the expectation the humeri of raccoons would feature a more dense trabecular matrix, with higher BV/TV, TbTh, TbN, and DA than the femur, to support this increased load, especially considering the smaller size and cortical density of these forelimb elements compared to the hindlimb. However, we observe the opposite pattern in raccoons, with nearly all of these features significantly higher in the hindlimb, and TbN relatively comparable. Both the humerus and femur appear to be developing larger and thicker trabecular struts over time to support against higher strain even as TbN decreases, but the femur does so to a greater extent. TbN being the only character to not show significant difference between the humerus and femur after 2 months suggests that the TbN within the articular heads of long limb elements is more strongly impacted by the overall mass of the animal than by the disparate strains experienced by the limbs. This disparity in limb elements may be driven by the fact raccoons engage in a number of hindlimb dominated and exclusive behaviors that place a significantly higher strains on the hindlimbs compared to other fully quadrupedal mammals (MacClintock and Thomas 1981; McClearn 1992). The impact of these specific behaviors could be further tested by sampling a wider range of raccoon populations. Individuals in regions of the Americas with less tree coverage may utilize hindlimb arboreal climbing less than their relatives from the more forested Great Lakes region, with that difference in behavior reflected in less dense and more isotropic femoral trabeculae. Future testing may also determine if these trends are present among “traditional” quadrupedal mammal species, which would imply that trabecular features may be impacted more by the overall size of the surrounding bone instead of the magnitude of forces applied to it.

### Trabecular orientation

Unlike the univariate trabecular metrics, which show a gradual shift in structure across ontogeny, primary trabecular orientation for both the humerus and femur can be divided into two distinct arrangements with clear divides between age groups. The struts arranged horizontally along the anterior/posterior axis in the youngest individuals rapidly undergo a shift to a more uniformly vertical orientation by the end of an individual's first year. This abrupt transition appears to be caused by several factors. The full ossification of hyaline cartilage that comprised much of the articular head at 2 months ensures that loading strain exerted on the joint is no longer deflected laterally and instead bears down vertically on the structure in the direction of gravity. The infrequent and short excursions outside the den that 2-month individuals can undertake increase in duration and distance with age, until individuals are engaging in foraging, walking, and climbing behaviors that exert far greater strains on the limbs. This increase in stress helps to direct the trabeculae to align in a uniform vertical orientation, parallel to the force of gravity. Finally, this progression is further facilitated by an increase in vascularization within the bones during secondary endochondral ossification, as blood vessels invade the still developing epiphysis to transport osteoclast and osteoblast cells. These cells facilitate the growth and development of trabecular struts and allow the bone to more rapidly react to strains produced during locomotion (Streeter 1949; Charbord et al. 1996).

Although this distinct shift from juvenile to adult orientations is present in both the humerus and femur, the rates at which this transition occurs vary between the two elements. Specifically, by 4 months the orientations of trabeculae within the humerus have been reoriented to the adult configuration, whereas this process lags in the femur until a full year has passed. The 4–12-month age range features femora with a trabecular azimuth with less variance than their younger peers, but it still retains a primarily horizontal plunge aligned parallel to the sagittal plane. Though we might expect a further gradual transition with age, by 14 months the trabecular orientations have progressed to the same vertical structures present in the humerus some 10 months earlier.

The causes of this transitional lag between the humerus and femur are unclear, and many of the factors that influence trabecular development and modification cannot be used as sole explanations for this phenomenon. Differences in loading strain between the humerus and femur likely match those present in other mammal species during quadrupedal motion, with the humerus enduring higher substrate reaction forces. However, the utilization of hindlimb-dominated behaviors in raccoons, and the presence of larger more uniform trabecular struts in the femur suggest bipedal locomotion may have a stronger impact on trabecular development. Additionally, the timing and frequency of these behaviors in young raccoons further complicates matters. Although these high-stress behaviors are not frequently observed in 2-month-old individuals who are still working on getting their bearings, they are frequently observed by 4–6 months, when juveniles are far more independent. The impact of strain on trabecular structures can be influenced by several factors, but previous work has demonstrated that an increase in both strain magnitude and frequency have a significant impact on BV/TV and strut orientation (Keaveny et al. 1999; Judex et al. 2007; Ozcivici et al. 2007; Barak et al. 2011). A possible explanation may be that before leaving the den, juvenile raccoons have ample time for their humeral trabeculae to adjust to stresses exerted during quadrupedal locomotion before incorporating more hindlimb-dominated movements. Nevertheless, it is unclear if the very sedentary lifestyle of very young raccoons provides enough activity to stimulate humeral trabecular development, and more testing will need to be done to quantify these transitions.

Puberty, and the resulting release of hormones, has also been demonstrated to influence the development of trabecular structures. Several studies have demonstrated the use of hormonal antagonists to delay puberty in females can retard the growth of trabecular struts oriented in the direction of primary loading (Georgopoulos et al. 2001; Yingling et al. 2007). Additionally, both oestrogen and testosterone have been suggested to be integral in the bone formation and resorption process that creates trabeculae (Finkelstein 1996; Seeman 2002). It can therefore be inferred that the production of these reproductive hormones during puberty has a significant effect on the trabeculae developing during endochondral ossification. However, these studies have shown no evidence to suggest the presence or absence of these hormones has a variable effect on trabeculae in different regions of the body. The orientation shift of humeral trabeculae occurs during the raccoon puberty window of 3–12 months, whereas femoral trabeculae still retain comparable orientations to younger, pre-puberty individuals. The production of hormones helping to influence trabecular growth would be expected to have a uniform effect between the humerus and femur and cannot account for the lag present during puberty.

Further studies focusing on ontogenetic changes in trabecular orientations within long-limb elements are needed to assess whether any of the above factors or other, unknown factors influence the transition in orientation across ontogeny with variable effects on different bones. Additionally, future research should also investigate quadrupedal species outside of the genus *Procyon* to determine if this trend is consistent in other taxa, or exclusive to raccoons.

## Conclusion

*Procyon lotor* undergoes a significant change in trabecular architecture of both the humerus and femur through the course of an individual's life, with a rapid change occurring during the 8-month span over which puberty occurs. These changes trend toward a decrease in TbN and density as the space between struts increase, necessitating a higher percentage of struts to orient vertically to preserve internal support against strain exerted during generalist locomotor behaviors. Although these overall trends are consistent between the humerus and femur, the rate and extent of their changes vary significantly between the two bones. Several of these differences cannot be explained currently and additional research on the driving forces of trabecular remodeling during puberty is needed to resolve these issues. Additionally, although *P. lotor* exhibits a generalist body plan that is comparable to a myriad of mammalian species, further research will need to be conducted to analyze if the trends seen in the species are consistent across other generalized and more specialized taxa.

Key differences in the ontogenetic development of BV/TV and DA in raccoons compared with humans and other primates highlight a previously unexplored disparity in trabecular structures. With the specific factors driving these differences still unclear, there is a need to sample additional taxa to quantify this variability and to understand the extent to which phylogeny, ecomorphotype, and posture influence trabecular development. Quantifying this diversity will elucidate how various species develop trabecular structures to support differing loading strains, and if those structures can be used to predict behavior in older, fossilized species.

The differences between the fore and hindlimbs also highlight the limitations in previous meta-analyses that combine distinct skeletal regions to bolster the size of datasets for meaningful statistical analysis. The overall trabecular growth trends can be similar between disparate bones, but the distinctions are wide enough in our work that we caution future researchers looking to combine elements in their research.

## Supplementary Material

obae038_Supplemental_Files

## Data Availability

Binarized tiff stacks of trabecular and hollow space for all measured elements are available on Dryad.
