# Supplementary material for: Raccoons Reveal Hidden Diversity in Trabecular Bone Development
Source: Integr Org Biol. 2024 Oct 21;6(1):obae038. doi: 10.1093/iob/obae038 (PMC11495488; doi:10.1093/iob/obae038)
Supplement: obae038_Supplemental_Files [file obae038_supplemental_files.zip › Tristan Reinekce Journal Integrative Organismal Biology Supplementary 3.docx]

Figure S3A


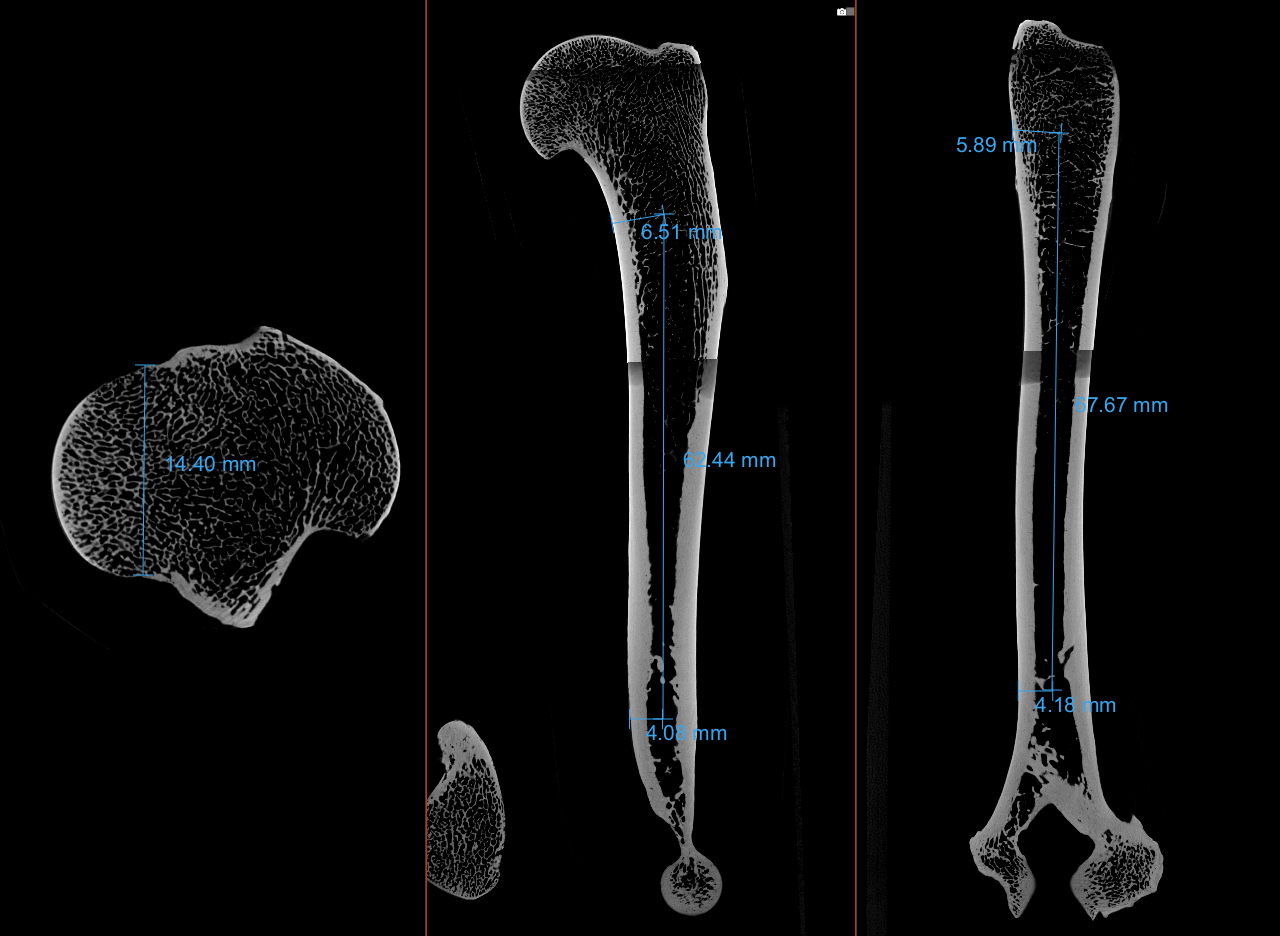


- Bones are isolated and oriented such that the midpoint of the proximal and distal metaphysis are aligned along the Z-axis. Once the bone has been made vertical, the widest points on either end of the articular head are aligned such that the resulting line rests on the Y-axis, and the articular head faced the -X direction.

Figure S3B


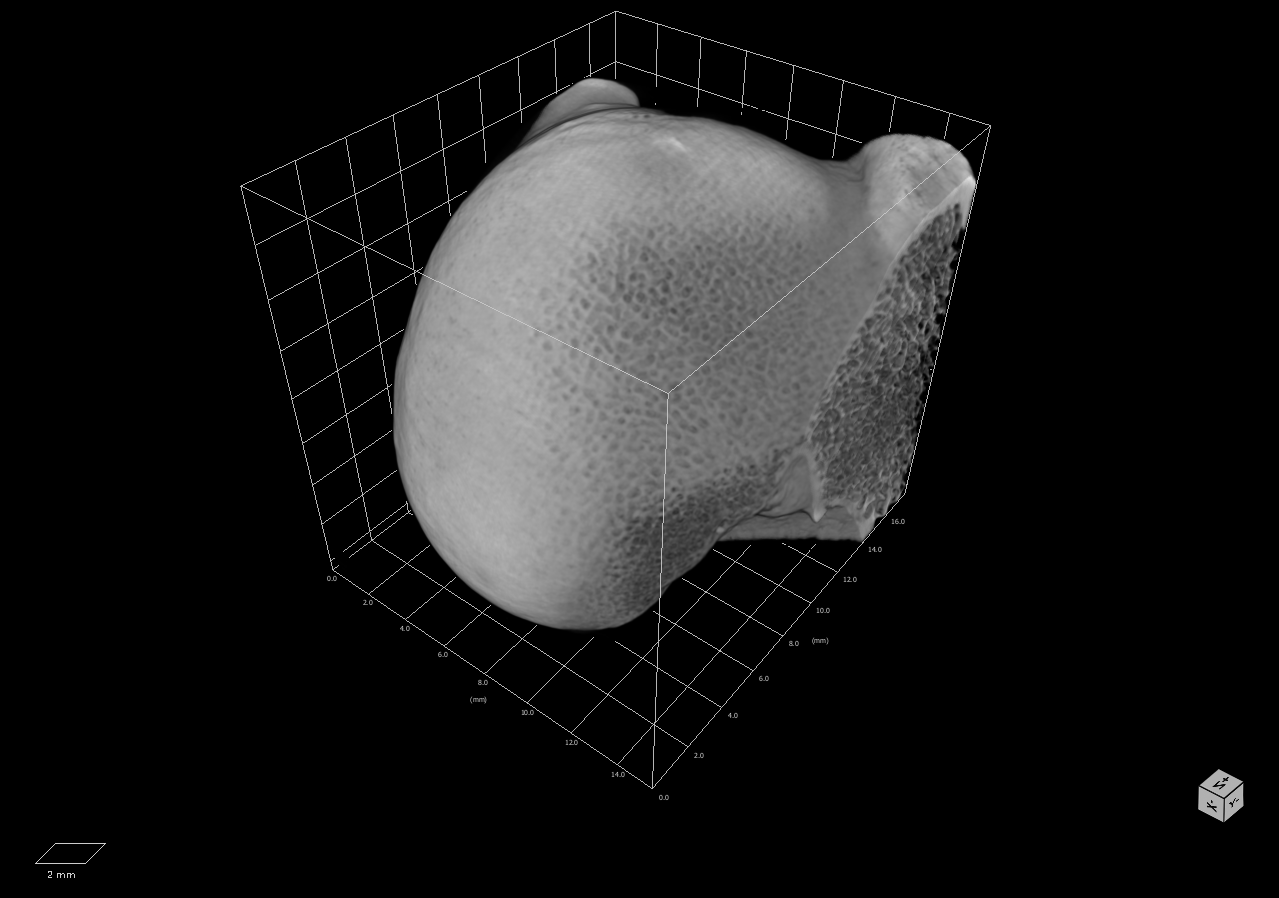


- Once the bone has been oriented, a cubic clipping box is drawn around the articular head to crop it from the rest of the bone.

Figure S3C


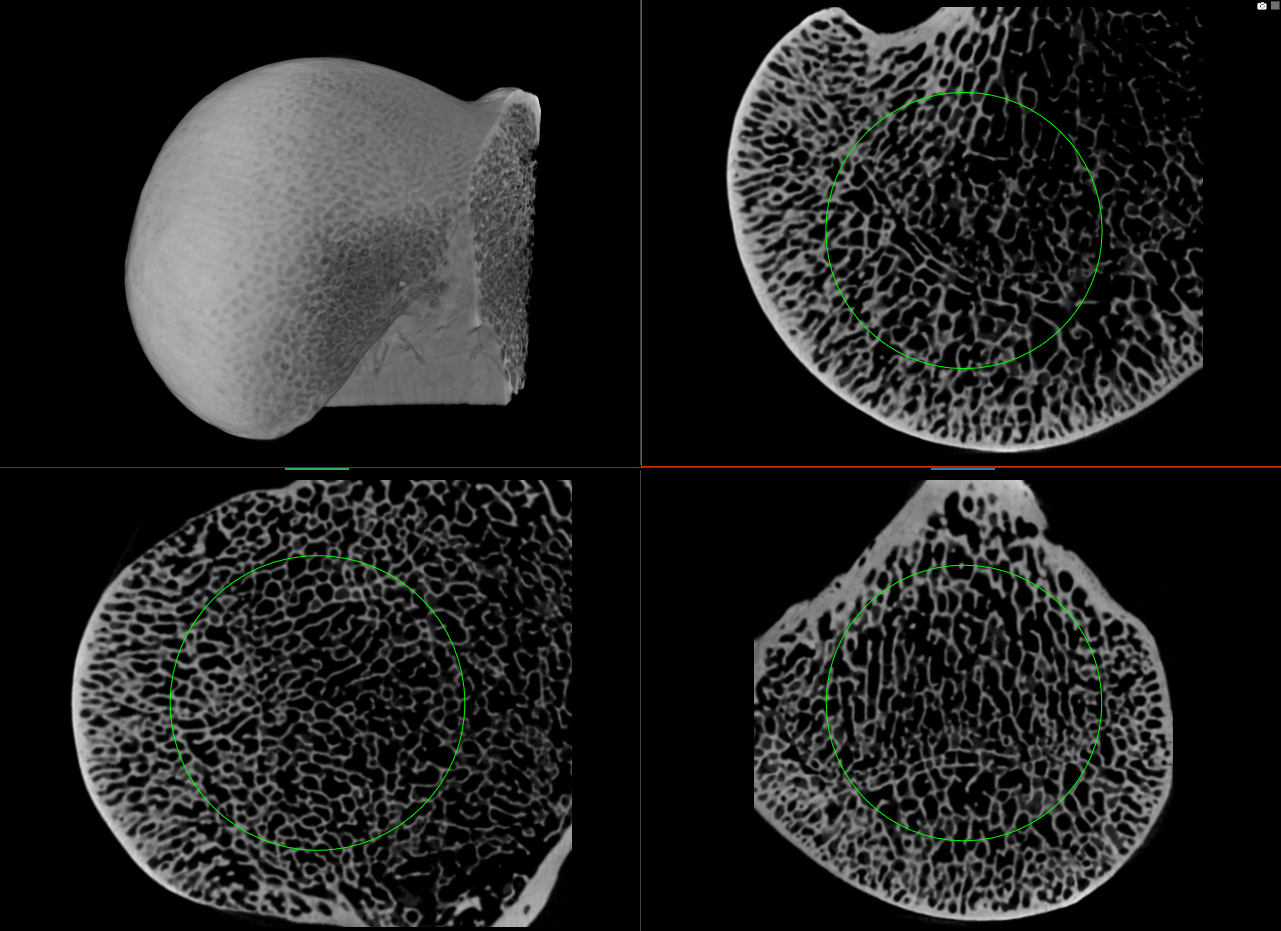


- From this isolated articular head, Dragonfly’s “Sphere” shapes tool can be used to place a sphere in the center of the articular surface. The sphere is then expanded or contracted to maximize the amount of sampled trabecular bone with excising any cortical elements.

Figure S3D


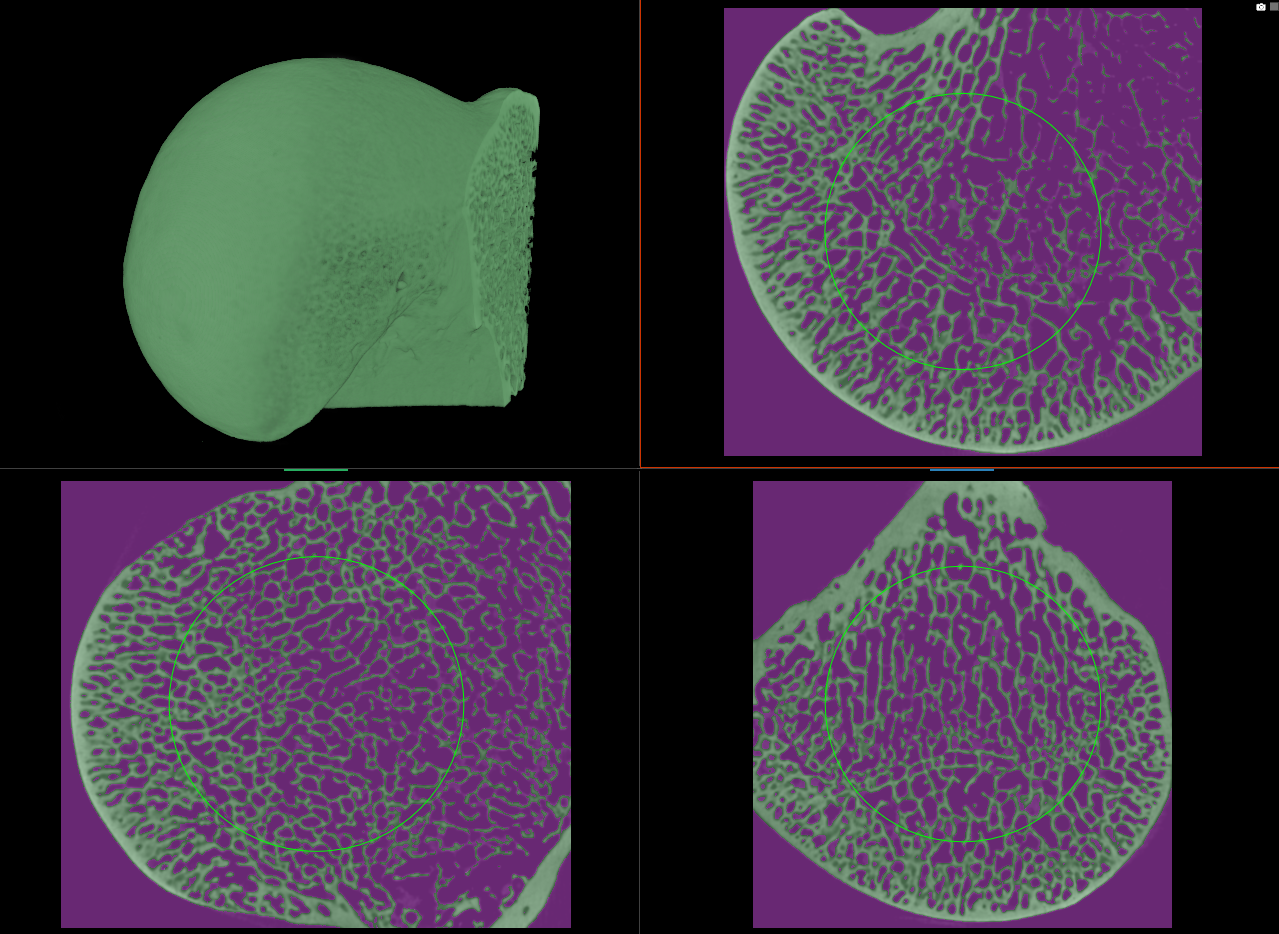


- From Dragonfly’s “Segment” tab, the otsu sorting algorithm is used to isolate and select the trabecular bone from the surrounding space. This trabecular ROI is also inverted to select the non-trabecular space

Figure S3E


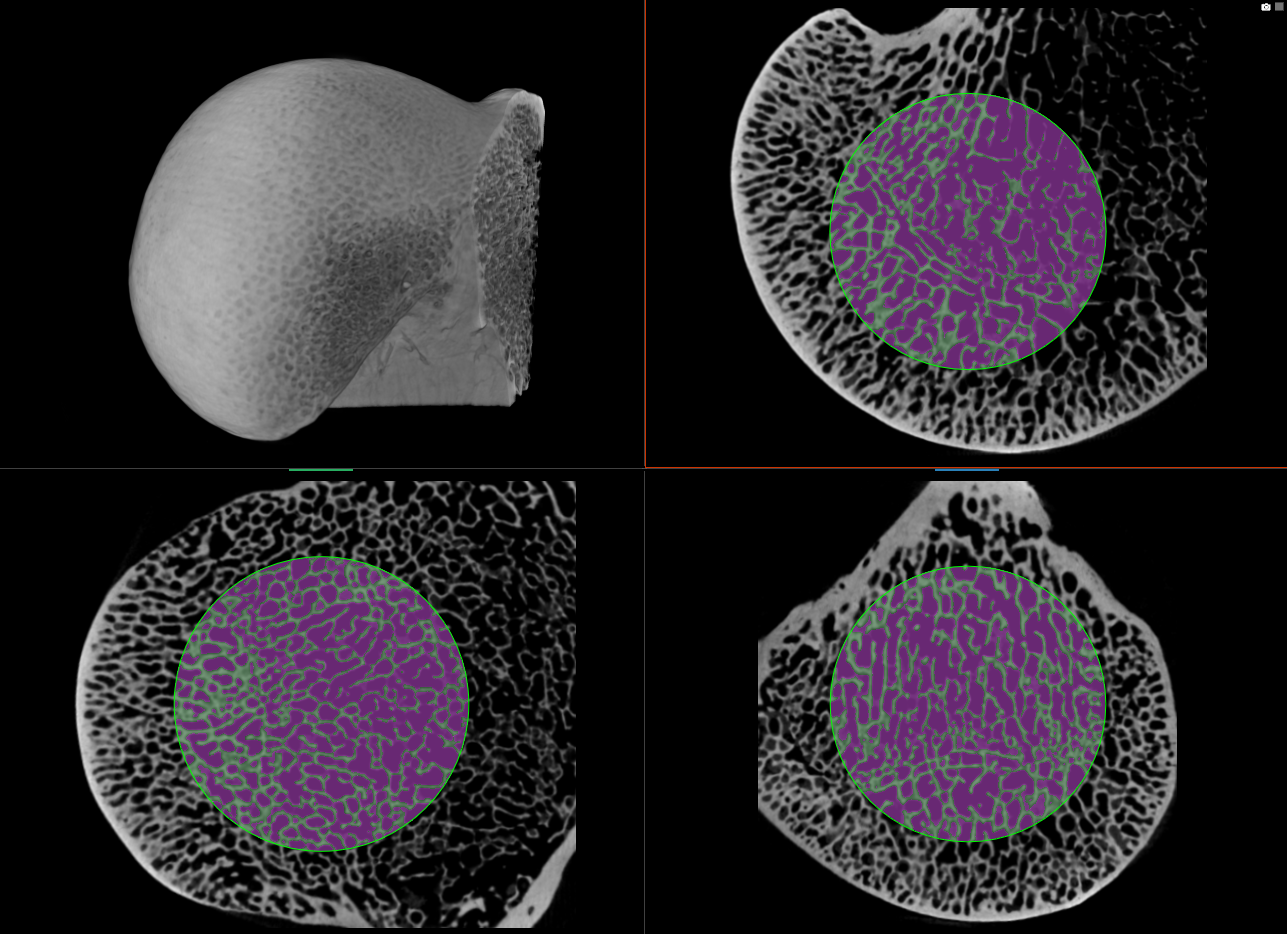


- A specific section of both the trabecular and non-trabecular ROI is selected using the sphere tool, before it is binarized and exported for analysis in other programs. The volume of both of these cropped ROI is also recorded to determine BV, TV, and BV/TV
